# The impact of CDIO's dimensions and values on IT Learner's attitude and behavior: A regression model using Partial Least Squares

**DOI:** 10.1016/j.heliyon.2022.e11433

**Published:** 2022-11-04

**Authors:** Ahmed Shuhaiber, Monther Aldwairi

**Affiliations:** a College of Technological Innovation, Zayed University, Abu Dhabi, 144534, United Arab Emirates; b Computer and information Technology, Jordan University of Science and Technology, Irbid, 22110, Jordan

**Keywords:** CDIO, Crowdsourcing, Gamification, Information security learners, Regression analysis, SEM-PLS

## Abstract

CDIO (Conceiving-Designing-Implementing-Operating), crowdsourcing and gamification are gaining more popularity in IT education. However, factors that influence learners' attitude toward this method are yet to be discovered. Therefore, this study aims to develop and test a conceptual model of implementing CDIO-based curriculum in IT education. For this purpose, CDIO dimensions were conceptualized and developed into questionnaire items. Then 141 students who experienced the CDIO method in information security course and lab, were sampled through action-research approach to investigate their perceptions and experiences about the learning stages, dimensions and values of this teaching method. Data gathered were analyzed by multiple regression algorithm using Partial Least Squares-Structural Equation Modeling (PLS-SEM) statistical approach. The results reveal that the ‘mastery of the concept’, ‘implement and operate’, ‘perceived values’, ‘demonstration and resources’, and ‘admin’ could significantly (in direct and indirect paths) affect learner's intention to accept the CDIO method and adopt it in IT classes. Finally, implications to theory and practice were indicated, and future research directions were suggested.

## Highlights

• CDIO methodology is a practical gamification approach to teach IT and Engineering courses.
• Still, no CDIO-based evidence that shows the learners' attitudes towards and acceptance of this method.
• A novel model of the CDIO dimensions is developed, validated and tested statistically using PLS-SEM approach.

## 1 Introduction and previous studies

The advent of technology, the wide-spread and penetration of smart phones' devices, and the virtual reality technologies are revolutionizing the field of education. Today's generations Z and Alpha form most of the students in colleges and schools. The different mindsets and new skills of browsing, searching, texting, digesting snippets of information, gaming and watching videos, prompted educators to reconsider their educational methods [1]. More recently, the Covid-19 pandemic disrupted most of the of normal life aspects. The pandemic forceful lockdowns and social distancing measures posed new challenges and prompted many to rethink how education of future generations will press forward [2]. It started a kerfuffle over the delivery methods. Therefore, education will no longer be bound to brick-and-mortar buildings and online distance learning is becoming the norm. Technology will continue to play a vital role in reshaping education and will hold the keys for future pedagogical approaches. Hybrid educational systems that involve online learning, self-paced and instructor-led, autonomy and working in groups, theory and hands-on project-based learning, traditional assessment and innovative projects, and appropriate support and contingency planning are among the best candidates [3].

Information Technology (IT) and particularly information security courses are poised to lead the way and innovate new learning strategies to respond to the challenges. Only recently had universities introduced information security courses at the undergraduate levels. Patricia Logan in [4] identified two models of security education: (1) private certification bodies, and (2) proprietary industry courses. She also emphasized the importance of building skill sets, laboratory experiments, and legal aspects in information security management curricula. Yoon et al. in [5] used Protection Motivation Theory (PMT) research model to assess information security students' behaviours. The model studied perceived vulnerability, perceived severity, response efficacy, behavioural intentions security habits effects on information security behaviours. They surveyed 202 students and concluded that information security practice is correlated to levels of severity, and that student attitudes are affected by their information security education. The survey had limited audience in terms of majors leaving no room for different attitudes to be measured. The study, however, was not concerned with the teaching methodologies and learning models’ effects on information security behaviours and attitudes.

One of the common concerns in IT courses is the ability of the students to apply what they have learned in realistic scenarios. Carol Hsu applied situated learning strategy in a “Security of Information Systems in Organisations” course in the UK [6]. The hybrid online and face-to-face security course included: lectures, guest seminars, group tasks, essay and the use of an online systems. The case study subjected 36 MSc. students to semi-structured interviews, survey and monitoring of their online activities. Thirty of the initial questionnaires were used and the study concluded that the students exhibited high levels of engagement with industry experts in the guest lectures. Additionally, they faced problems working together on group assignments although reportedly the quality of team assignments trumped individual assignments. Finally, very thorough, and analytical students’ discussions were carried online. However, the study main limitation was that it failed to capture the real-world settings, and the course was not of a technical nature. Guest lectures by industry experts hardly mimics working in a stressful and real time information security incident response situation. We believe the sample size was very small, the analysis was lax, and the attitudes/behaviours were not properly measured.

It worth mentioning that relevant research studies focused more on the gamification concept than the CDIO concept. For example, the results of this study [7] indicated that gamification in engineering classes can influence student's academic achievement by motivating and engaging them in the ‘operations research’ topic. Also, the same study indicated that gamifications in classrooms has social impacts on the student's behaviour. Another study [8] that studied the main gamification concepts in 50 articles and found that some elements should be incorporated in gamification such as points, leaderboards and badges, in order to improve engagement with learners. In another study, two educational methodologies: gamification and flipped classes were employed in a quasi-experimental approach and found to have positive impacts on learners' academic performance based on their age category [9]. In sum, education is undergoing a methodological transformation, towards achieving better learning outcomes and qualities, and given that CDIO approach incorporates more dimensions of gamification and crowdsourcing, it should be getting more attention in high education.

The CDIO framework, which stands for Conceive – Design – Implement – Operate, has been popular in teaching IT courses. The teaching by doing framework has gained a lot of attention recently in IT courses [10]. Guo and Yan introduced CDIO into computer major to overcome the traditional Chinese educational system bias towards theory. Their goal was to bridge the gap between the sills attained in class and those required by the software industry. The focus was on learning by doing and project-based learning [11]. Bin and Shimming applied CDIO in a software testing course to be more student centred. They redesigned the syllabus to be more problem-based and project-based learning. In the course, theory represented 35%, experiments 15% and the course project 50%. Therefore, rather than the instructor explaining the knowledge, the focus was on doing and implementing the skills to attain that knowledge. Rather than traditional laboratories they utilized ‘occupation experience centres’ equipped with private meeting rooms and the latest software testing tools [12]. Song et al. applied CDIO in a web design course were gamification and crowdsourcing were also deployed. No regression or equivalent analysis was used to link the teaching methods to perceived enjoyment, attitudes or behaviours [13].

Information security courses and ethical hacking per say are unique in that a comprehensive set of skills must be developed and, in the fact, that the tools and equipment needed carry a lot of risk. Some of the tools are plain illegal and the experiments performed might result in leakage of malware, intrusion incidents and committing of illegal activities [14]. The attacks used are real, the threats are consequential and mitigation techniques are necessary [15]. Yu and Wang proposed CDIO tightly coupled lab experiments and theory in a network security class. The focus was improving the students’ hands-on skills, network design abilities and soft skills. That was achieved by designing security simulation labs with network security, hacking and anti-hacking tools. It was not clear how the efficacy of the approach was evaluated, as no measurement instrument was used, but the authors claimed unprecedented motivation among participants and solid skills set attainment [16]. A hacking exposed course using CDIO was ran at Duy Tan University. The course included lecturing, in class discussions, out-of-class research and short 3–5 days projects. What was striking is that the students practiced hacking on production websites, which can the least be said as unethical and borderline criminal! No formal evaluation or measurement instrument was used other than “observation by the instructor” and they found that “overall quality of students had been improved” [17].

We proposed and designed a CDIO-based information security and ethical hacking courses. The hacking life cycle was mapped onto the CDIO framework in an iterative fashion. The course included reduced in class instruction (theoretical knowledge dissemination), student teams, practical in class activities, assignments, and limited testing as well as gamified final project. In addition to mapping the hacking process onto the CDIO framework, the course had the following contributions [18].

1. Ungraded in class activities that are instructor-led hacking exercises. Basically, walking the students through hacking numerous vulnerable machines. This helped in skills building, developing an arsenal of diverse attack tools, and engrave the hacking life cycle in the student's memory. The vulnerable machines are designed specifically to teach different attack vectors and tools.
2. Eliminated the final test as the main assessment because the learning outcome is measuring the skills developed during the semester rather than what the student had memorized.
3. Introduced gamified final project, as an alternative fun assessment. This was the final learning by doing exercise that took the form of capture the flag competition between the different student teams. The project was conducted live with an online scoreboard showing the teams progress in real time.
4. The project and in class activities encouraged Bring Your Own Device (BYOD), use of Internet competitive intelligence and collaborative problem-solving. Online and offline inter- and intra-team collaboration were encouraged in a crowdsourced fashion.

It is worth noting that all the experiments and activities are performed in an isolated lab, separate from the university production network. All the victim machines are local virtual boxes, and no real systems or networks were jeopardized during the course. One hundred and forty-one students were surveyed over three semesters. The research model items in the questionnaire were analysed by using the method of Partial Least Squares in Structural Equation Modelling (SEM-PLS) approach. CDIO lab experiments helped build the students' skills and had a profound effect on perceived enjoyment of the course. The perceived enjoyment in turn made the courses more desirable, affecting the attitude towards the course and consequently affecting the student's intentions to take similar courses. The gamification aspect helped achieve the learning outcomes in a fun way reflecting on the positive relationship of the students and the university as a whole [19].

## 2 Research methodology

The current study employed a quantitative approach to examine the learners’ perceptions and experiences about the CDIO implementation in information security classes. To achieve this, learners were asked to complete a questionnaire survey by the completion of the CDIO-driven class. The questionnaire instrument included questions about the dimensions of the CDIO (mastery of concepts and design, and implement and operate), perceived values of CDIO classes, CDIO demonstration, resources and administration availability by the instructor, attitude towards the method and behavioural intention. A Likert scale of five-point was used to measure the research variables, ranging from 1 to 5 with the values from strongly disagree to strongly agree, respectively. The survey instrument was improved during a pre-test by a panel of academics to ensure face validity.

As for the sampling approach, this study followed a non-random technique to approach the targeted sample, which is the convenience approach. Links of online surveys were shared with the learners using the university e-learning platform. In total, six lab classes at the College of Technological Innovation were targeted to implement the CDIO technique at Zayed University in its two campuses: Dubai and Abu Dhabi. As a result, 141 senior students participated in this study during a twelve-months period. All responses were checked and validated as complete and valid for analysis.

For ethical considerations, the researchers included a consent form and information to participants in the beginning of the survey, in order to familiarize the participants with the goal of the study, the voluntariness nature of the participation, the anonymity and privacy of the participants' identities, the length of the survey (7–10 min), the confidentiality of research data (to be stored at the researchers’ database), the research findings (as part of conference or journal publications) and other details.

Regarding the data analysis, the researchers conducted two types of statistical analysis: descriptive statistics that were performed by using SPSS statistical software to describe some research items, and SEM-PLS statistical analysis, which was performed by using SmartPLS 3.0 software to test the research model. SEM is a set of statistical models, which explains the relationships among multiple variables, and gives a holistic picture of the entire model by showing the connections among the variables [20]. As one popular approach of SEM; PLS is preferred for a complex theory testing and causal-predictive analysis, especially when small samples are employed [20, 24]. Also, PLS can be applied to complex structural equation models with many constructs [20, 24]. Therefore, employing SEM-PLS in this research was considered appropriate to test the proposed research model. The following section demonstrates the research findings.

## 3 Research findings

The subsequent sections demonstrate the findings of the descriptive statistics and the SEM-PLS analysis, in its both outer and inner model testing steps.

### 3.1 Descriptive analysis

This subsection includes some descriptive statistics about students’ demographics, the clarity of the new lab structure and to what degree the students agreed that the course was delivered as proposed in the course syllabus and were calculated by the software SPSS27.0. Firstly, the targeted sample in this research includes students from both male and female students. In details, the female accounted for 61.3% of the total students, whereas male students accounted for only 38.7%, while the total number of students who participated in this research was 141. All participated students were senior students (in their 4th and last year of study in their degree).

The students were asked about whether the new lab structure was clearly explained at the beginning of course. As a result, most of the students (88.7%) agreed/strongly agreed that the CDIO lab structure was explained clearly, as shown in Table 1.

**Table 1:** The CDIO lab structure.

		Frequency	Percent	Valid percent	Cumulative percent
Valid	1	4	2.8	2.8	2.8
	3	11	7.7	7.8	10.6
	4	47	33.1	33.3	44.0
	5	79	55.6	56.0	100.0
	Total	141	99.3	100.0	
Total		141	100.0		

In addition, the students were also asked about whether the course was delivered as outlined in the course syllabus and followed the new structure of the CDIO practice. Consequently, the vast majority (almost 90%) of the students agreed that the instructor adopted the new structure of the lab that adopted the CDIO implementation. Further statistics about this point are presented in Table 2.

**Table 2:** The delivery of the CDIO lab.

		Frequency	Percent	Valid Percent	Cumulative Percent
Valid	1	4	2.8	2.8	2.8
	2	2	1.4	1.4	4.3
	3	14	9.9	9.9	14.2
	4	63	44.4	44.7	58.9
	5	58	40.8	41.1	100.0
	Total	141	99.3	100.0	
Total		141	100.0		

The descriptive statistics of each item in the survey show the minimum and maximum scores, the range, the mean, and the standard deviation. As shown in Table 3 below, it is noteworthy that MCD2 has the highest mean scores (4.16), and that the associated construct with this item (Mastery of Concept and Design) also has the highest mean score among other variables. On the other hand, the item PV4 has the lowest mean score of 3.68. All scores have a minimum score of 1 and a maximum score of 5. In addition, almost all the standard deviation scores are around the value of 1. More details could be found in Table 3, and the items wording can be found in the Appendix.

**Table 3:** Descriptive statistics of the survey questions.

Item	N	Range	Minimum	Maximum	Mean	Std. Deviation
PV1	141	4	1	5	4.01	1.086
PV2	141	4	1	5	3.91	1.066
PV3	141	4	1	5	3.87	1.064
PV4	141	4	1	5	3.68	1.110
PV5	141	4	1	5	3.92	1.008
PV6	141	4	1	5	3.94	1.081
PV17	141	4	1	5	3.77	1.136
MCD1	141	4	1	5	4.13	1.027
MCD2	141	4	1	5	4.16	.973
MCD3	141	4	1	5	3.91	1.027
MCD14	141	4	1	5	4.14	.953
IO1	141	4	1	5	3.93	.990
IO2	141	4	1	5	3.98	.982
IO3	141	4	1	5	3.99	.922
IO4	141	4	1	5	4.07	.968
IO5	141	4	1	5	3.96	1.041
RD1	141	4	1	5	3.87	1.020
RD2	141	4	1	5	3.86	1.004
RD3	141	4	1	5	4.05	.966
Demo1	141	4	1	5	3.91	1.048
Demo2	141	4	1	5	3.94	.977
Iskill1	141	4	1	5	3.97	1.102
Iskill2	141	4	1	5	3.93	1.119
Iskill3	141	4	1	5	3.94	1.126
Tskill1	141	4	1	5	4.02	1.065
Tskill2	141	4	1	5	3.81	1.062
Tskill3	141	4	1	5	3.84	1.030
Tskill4	141	4	1	5	3.72	1.071
Tskill5	141	4	1	5	3.88	1.072
PE1	141	4	1	5	4.11	.924
PE2	141	4	1	5	4.09	1.032
PE3	141	4	1	5	3.91	1.105
Att1	141	4	1	5	3.82	1.051
Att2	141	4	1	5	3.98	1.017
Att3	141	4	1	5	3.80	1.191
Att4	141	4	1	5	4.00	1.069
BI1	141	4	1	5	3.96	1.058
BI2	141	4	1	5	4.02	1.024
BI3	141	4	1	5	3.99	1.042
Valid N (listwise)	141					

More advanced data analysis took place in order to understand the relationship among the research variables, and to investigate their impact on students’ attitudes and behaviours.

### 3.2 SEM-PLS analysis

As mentioned earlier, the PLS model is usually analysed and interpreted in two stages: (1) by assessing the reliability and validity of the measurement model (the outer model of constructs and items), and (2) by assessing the structural model through interpreting the path coefficients and identifying the adequacy of the research inner model [20]. The following sections discuss the results of these two stages, by using SmartPLS 3.0 software, which is specialized in performing the Structural Equation Modelling-Partial Least Squares analysis.

### 3.3 PLS measurement (outer) model results

Firstly, the PLS outer model is assessed by examining the scores of the item loadings, which are tested in order to examine the correlations between the latent variable and the reflective indicators. As a result, nearly all the items are found above the acceptable level of (0.6), thus demonstrating reliable items, with the exception of the highlighted item ‘Efficiency6’ in Table 1, and accordingly eliminated from the study. In total, 35 items of the survey are validated to measure the dependent and independent variables, as shown in Table 4.

**Table 4:** Item loadings.

	Attitude	Behavioral intention	Demo	Implement & operate	Mastery of concept	Perceived values	Resource and admin
ATT1	0.883						
ATT2	0.903						
ATT3	0.9						
ATT4	0.913						
BI1		0.912					
BI2		0.934					
BI3		0.944					
Demo1			0.93				
Demo2			0.933				
IO1				0.913			
IO2				0.938			
IO3				0.898			
IO4				0.829			
IO5				0.914			
MCD1					0.909		
MCD2					0.923		
MCD3					0.862		
MCD4					0.809		
PV1						0.913	
PV2						0.818	
PV3						0.831	
PV4						0.843	
PV5						0.853	
PV6						0.873	
PV7						0.881	
RD1							0.905
RD2							0.879
RD3							0.845

Another measurement of the item's quality is testing the construct validity, which assesses whether the measures chosen are true measures of the constructs and represent the associated constructs [20]. Construct validity is usually established by examining both convergent and discriminant validity. Convergent validity is the extent to which a construct correlates with its measures [20] and is demonstrated when the Average Variance Explained (AVE) score exceeds or equal to 0.5 [20, 21]. As shown in Table 5, the AVE scores for all constructs are exceeding the cut-off point of 0.5, which demonstrates convergent constructs. Alternatively, convergent validity could be assessed by examining the constructs' scores of the composite reliability [21]. As a result, all constructs demonstrate high scores of composite reliabilities by exceeding the .60 cut off point [20].

**Table 5:** Reliability and validity estimates.

	Cronbach's alpha	rho_A	Composite reliability	Average variance extracted (AVE)
Attitude	0.922	0.924	0.944	0.81
Behavioral intention	0.922	0.924	0.95	0.865
Demo	0.847	0.847	0.929	0.867
Implement & operate	0.94	0.942	0.955	0.808
Mastery of concept	0.899	0.901	0.93	0.769
Perceived values	0.941	0.942	0.952	0.739
Resource and admin	0.851	0.875	0.909	0.768

In addition, Cronbach's alpha measures are examined to assess the internal consistency of the constructs and is achieved when the reliability estimates are greater than .70 [20, 22]. As presented in Table 5, all scores exhibit high reliability estimates, with Cronbach's alpha coefficients exceeding the .70 cut off points [20, 22], thereby, satisfying the second requirement of convergent validity. Overall, all variables in this study demonstrate valid and reliable constructs. Moreover, the researchers examine the discriminant validity and intercorrelations across constructs, which shows that the square root of the AVE scores of each variable is greater than any correlation between that constructs and the others, which demonstrates the discriminant validity of all constructs, as shown in Table 6.

**Table 6:** Discriminant validity and constructs’ intercorrelations.

	Attitude	Behavioral intention	Demo	Implement & operate	Mastery of concept	Perceived values	Resource and admin
Attitude	0.9						
Behavioral intention	0.882	0.93					
Demo	0.641	0.675	0.931				
Implement & operate	0.801	0.776	0.716	0.899			
Mastery of concept	0.757	0.75	0.694	0.83	0.877		
Perceived values	0.736	0.693	0.589	0.767	0.7	0.859	
Resource and admin	0.731	0.721	0.721	0.775	0.773	0.752	0.876

PLS structural (inner) model results.

**Figure 1:**
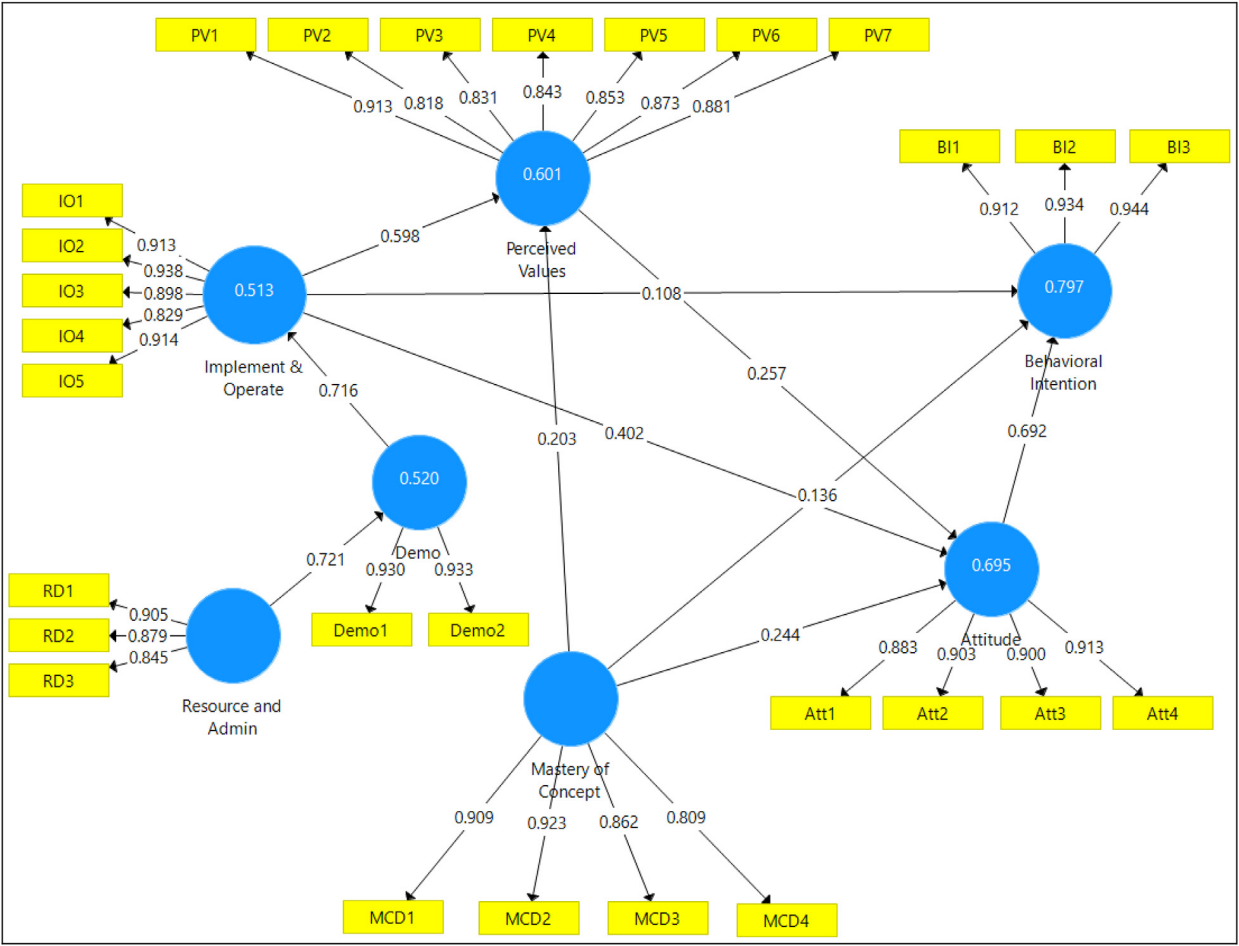
Research model.

Secondly, the PLS inner structural model is examined to assess the significance of the regression paths and the predictive power of the model. Table 7 highlights the Beta values of each the latent variables, T-Statistics, P-Values and hypotheses results [23]. As a result, all hypotheses are supported in 0.05 significant level. Figure 1 shows the conceptual model as tested in the SmartPLS3.0 software.

**Table 7:** Results of inner model testing.

	Original sample (O)	T Statistics (|O/STDEV|)	P Values	Hypothesis result
Attitude → Behavioral Intention	0.692	10.391	0.000	Supported
Demo → Implement & Operate	0.716	11.802	0.000	Supported
Implement & Operate → Attitude	0.402	3.176	0.002	Supported
Implement & Operate → Behavioral Intention	0.108	0.964	0.336	Not Supported
Implement & Operate → Perceived Values	0.598	4.357	0.000	Supported
Mastery of Concept → Attitude	0.244	2.063	0.040	Supported
Mastery of Concept → Behavioral Intention	0.136	1.451	0.148	Not Supported
Mastery of Concept → Perceived Values	0.203	1.457	0.146	Not Supported
Perceived Values → Attitude	0.257	2.351	0.019	Supported
Resource and Admin → Demo	0.721	12.611	0.000	Supported

Given the model fit indices in Table 8, it is found that the Standard Room Mean Square (SRMR) is 0.081, which is in the range of 0.1 and 0.08 [20], demonstrating a good fit model. Also, another measure of the model fit, which is the NFI (Normed Fit Index), which has the score of 0.758. The closer the NFI score to 1, the better the fit, and thus the score of 0.758 is considered near to an acceptable fit of the model. Other model fit measures are shown in Table 8, below.

**Table 8:** Model fit results.

	Saturated model	Estimated model
SRMR	0.057	0.081
d_ULS	2.502	5.081
d_G	2.321	2.552
Chi-Square	1,722.48	1,768.51
NFI	0.764	0.758

## 4 Discussion

By testing the research model, seven hypotheses out of ten are found significant. It is noteworthy that the path with the highest impact in the current study is the influence of “Demo” on CDIO “Implementation and Operation” (β = 0.716, t = 11.802, P-value = 0.00), which then significantly impacts learners' attitude towards accepting CDIO learning method (β = 0.402, t = 3.176, P - value = 0.002). Attitude, in turn, significantly and directly influences learners' intention to practice the CDIO method for information security courses (β = 0.692, t = 10.391, P-value = 0.00). Other strong paths are the ones associated the impact of “Mastery of Concept” and “Perceived Values” on learners' attitudes towards the CDIO method (β = 0.244, t = 2.063, P-value = 0.02) and (β = 0.257, t = 2.351, P-value = 0.019), respectively. In addition, the impact of ‘Implementation and Operation’ on ‘perceived values’ and the impact of ‘perceived values’ on learners' attitudes towards the CDIO methodology are found to be significant (β = 0.598, t = 4.357, P-value = 0.00) and (β = 0.257, t = 2.351, P-value = 0.019), respectively.

The weakest path, however, is the influence of ‘Mastery of Concept’ on learners' Attitudes, which has the lowest score in affecting intention to use this methodology (β = 0.244), where it is still found to be significant on 5% confidence interval. Another weak path, though significant, is the influence of ‘Perceived Values’ on learners' attitudes towards applying the CDIO method in the classroom (β = 0.275, t = 2.351, P-value = 0.019).

The amount of variance explained by R^2^ is usually used, which provides an indication of the predictive ability of the endogenous variables. The R^2^ scores should be greater or equal to 0.10 [23]. As a result, the R^2^ value of ‘Behavioral Intention’ is moderate and equal to 79.7%, and 69.5% for ‘Attitude’ construct, and thus both endogenous variables show high amount of variance. Moreover, the two constructs ‘Perceived Values’ and ‘Implementation and Operation’ have moderate R^2^ scores of 60.1% and 51.3%, respectively. Overall, and since R^2^ scores are greater than 50% for the four endogenous variables, it is argued that more than the half of the observed variation can be explained by the model's inputs, which in turn provides another measure of a good fit model [20].

## 5 Conclusion

As a conclusion, the availability of the admin and educational resources can play an important role in presenting CDIO demonstrations, which in turn significantly influence the implementation and the operation of the method. The latter could impact learner's perceived values of the CDIO method, which directly influences their attitudes towards the method and then their intention to apply it. From another aspect, the mastering of concept would be a great start to impact learners' perceived values about the CDIO, as well as their attitude and behavior towards the method. Learners' perceived values about the CDIO are the key player in our model by presenting a mediating role among CDIO's dimensions (mastery of concept and design, implement and operate) and learners' attitude towards and intention to apply the CDIO in their undergraduate classes.

In summation, and in order to implement the CDIO methodology successfully in computer engineering and science classes, the adoption process of the CDIO should be organized and sequential. First, the CDIO context and syllabus outcomes should be clearly identified, aligned and integrated with the course syllabus. In each CDIO implementation, the practice should be enhanced by the instructor to reach the best practice and a possible innovation. Second, the lab structure should consider the CDIO aspects and consequently redesigned. Afterwards, the CDIO resources and facilities should be prepared and familiarized by the faculty who is required to develop his CDIO and course skills, and knowledge. Finally, the CDIO skills and course outcomes should be assessed and evaluated towards course, major operation and student learning.

**Theoritical and practical implications**

Theoretically, the crafted and validated research model contributes to the CDIO literature by presenting genuine relationships of the CDIO dimensions (mastery of concept and design, implement and operate) and learners’ attitude towards implementing the method. In addition, the results indicate the important role of resources and admin availability as well as demonstrating CDIO at the beginning of each class (if necessary), could not only impact the implementation and operation of the method, but also contribute to what values learners perceive the method. To the best of our knowledge, this aspect had not been discussed before.

For practitioners, instructors need to consider good preparation and administering of the tools and concepts associated with CDIO and provide a clear demo of how CDIO should be implemented in each class. Learners, form the other side, should understand clearly how the method works, what resources are required, and should be informed about all potential values associated with the method. In addition, it is highly recommended that instructors who plan to implement a CDIO-based class to follow the guidelines mentioned in the summary of the discussion part.

**Research limitation and future work**

There are some limitations associated with this study. For example, this research utilized surveys, which, by nature, are prone to measurement error and bias. To handle this, the researchers checked face validity with a panel of academics and established the convergent and discriminant validity and reliability of all research variables. Secondly, the convenience sampling approach chosen for this research is usually associated with result's generalizability to other subjects, subject bias, and less sample representativeness. The results of the current study could be limited to the information security classes and subjects selected for the study; however, our research results could extend the context of IT and Engineering classes to other classes, which follow the same description of the CDIO method as identified in literature, and build studying groups to develop CDIO concepts, implementation and operation is essential. As for future research work, we would suggest testing the model in different contexts and backgrounds, and for different schools and programs. Additionally, examining the results with the moderating role of gender or school, the role of culture, and the social influences through word of mouth among learners could provide a helpful insight to better understanding the CDIO acceptance factors. In addition, future work might include the examination of teacher engagement in some stages of applying CDIO in classrooms.

## Declarations

### Author contribution statement

Ahmed Shuhaiber: Conceived and designed the experiments; Analyzed and interpreted the data; Contributed reagents, materials, analysis tools or data; Wrote the paper.

Monther Aldwairi: Conceived and designed the experiments; Performed the experiments; Contributed reagents, materials, analysis tools or data.

### Funding statement

This research did not receive any specific grant from funding agencies in the public, commercial, or not-for-profit sectors.

### Data availability statement

No data was used for the research described in the article.

### Declaration of interest's statement

The authors declare no conflict of interest.

### Additional information

No additional information is available for this paper.

## APPENDIX

Perceived values

[PV1] I think the lab experiments were motivating to learn the subject.
[PV2] I think the learning pace of lab experiments was convenient.
[PV3] I think the lab experiments were well organized and prepared.
[PV4] I think the lab sessions were rich in terms of discussions and questions.
[PV5] I think the experiments were challenging to achieve better learning outcomes.
[PV6] I think the lab experiments support my independent learning.
[PV7] I think the lab experiments qualified me with the necessary skills required after graduation.

Mastery of concepts and design

[MCD1] I think the instructor explained the theory and concepts associated with the experiment very well.
[MCD2] I think the instructor explained the design and usage of different security instruments very well.
[MCD3] I think the in-class learning material help me to master the course concepts.
[MCD4] I think that the lab experiments improve my understanding of different security instruments.

Implement and operate in laboratory

[IO1] I think the concepts and design helped me in the implementation phase.
[IO2] I think the operate phase helped me better understand the concepts.
[IO3] I think the operate phase helped me improve my work.
[IO4] I think the lab experiments were highly relating to the course learning outcomes.
[IO5] I think the experiments and projects measured the course learning outcomes.

Resources and Administration

[RD1] I think the experiments were supported by adequate lab resources.
[RD2] I think the Blackboard resources for the course were useful to conduct the experiments.
[RD3] I think the lab technician provided adequate guidance on finding learning resources.

Demonstration

[Demo1] Tutors made demonstrations before I started with my experiments.
[Demo2] By completion, students demonstrated their finished projects and experiments.

Attitude towards the course

[ATT1] I like the way this course was delivered.
[ATT2] To me, this was a worthwhile class/lab.
[ATT3] I would like other courses to have similar lab setup.
[ATT4] Overall, I feel positive towards lab experiments.

Behavioral intention

[BI1] I intend to enroll in courses with lab experiments.
[BI2] I personally recommend this course to a fellow student.
[BI3] I will always try to enroll to similar method courses in my university life.
